# Advancing CAR T-Cell Therapy for Solid Tumors: Lessons Learned from Lymphoma Treatment

**DOI:** 10.3390/cancers12010125

**Published:** 2020-01-03

**Authors:** Aleksei Titov, Aygul Valiullina, Ekaterina Zmievskaya, Ekaterina Zaikova, Alexey Petukhov, Regina Miftakhova, Emil Bulatov, Albert Rizvanov

**Affiliations:** 1 Institute of Fundamental Medicine and Biology, Kazan Federal University, 420008 Kazan, Russia; altitov0@gmail.com (A.T.); aigul1692@mail.ru (A.V.); ekazmievskaya@gmail.com (E.Z.); alexeysakhalin@gmail.com (A.P.); regina.miftakhova@gmail.com (R.M.); 2 Laboratory of Transplantation Immunology, National Hematology Research Centre, 125167 Moscow, Russia; 3 Institute of Hematology, Almazov National Medical Research Center, 197341 Saint Petersburg, Russia; catherine3452@yandex.ru; 4 Shemyakin-Ovchinnikov Institute of Bioorganic Chemistry, Russian Academy of Sciences, 117997 Moscow, Russia

**Keywords:** chimeric antigen receptor, CAR T-cell therapy, TCR therapy, solid tumor, lymphoma

## Abstract

Chimeric antigen receptor (CAR) immunotherapy is one of the most promising modern approaches for the treatment of cancer. To date only two CAR T-cell products, Kymriah^®^ and Yescarta^®^, have been approved by the Food and Drug Administration (FDA) for the treatment of lymphoblastic leukemia and B-cell lymphoma. Administration of CAR T-cells to control solid tumors has long been envisaged as one of the most difficult therapeutic tasks. The first two clinical trials conducted in sarcoma and neuroblastoma patients showed clinical benefits of CAR T-cells, yet multiple obstacles still hold us back from having accessible and efficient therapy. Why did such an effective treatment for relapsed and refractory hematological malignancies demonstrate only relatively modest efficiency in the context of solid tumors? Is it due to the lucky selection of the “magic” CD19 antigen, which might be one of a kind? Or do lymphomas lack the immunosuppressive features of solid tumors? Here we review the existing knowledge in the field of CAR T-cell therapy and address the heterogeneity of solid tumors and their diverse strategies of immunoevasion. We also provide an insight into prospective developments of CAR T-cell technologies against solid tumors.

## 1. Introduction

The urgent need for novel approaches to the treatment of solid tumors is stipulated by cancer being the second most frequent cause of death worldwide. Solid tumors, such as lung, colon, prostate and breast cancer, are the major contributors to cancer mortality rates. Adoptive immunotherapy is one of the promising therapeutic modalities, potentiated with chimeric antigen receptor (CAR) T-cell therapy as its most prominent example. CAR T-cells have already revolutionized the treatment of relapsed and refractory (*R/R*) acute lymphoblastic leukemia (ALL), and В-cell non-Hodgkin’s lymphoma (NHL).

Furthermore, given the broad availability of appropriate targets, the application of CAR T-cell therapy now actively expands into the management of *R/R* multiple myeloma [1,2,3,4,5] and acute myeloid leukemia (AML) [6]. A phase I clinical trial of anti-CD123 CAR T-cells in AML reported three complete remissions (CR) and two stable disease (SD) cases in 12 infused patients without significant toxicity [7], while other clinical trials continue recruiting.

Solid tumors however, engage numerous mechanisms disrupting acquired immunity, and thus restrict the clinical potential of adoptive immunotherapy. Currently, the data on treatment of solid tumors with CAR T-cells are limited to several case reports or small phase I/II clinical trials [5]. The follow-up is often too short or not reported at all, thus making interpretation of treatment efficacy confusing and complicated. Nevertheless, the solid component is still significant in lymphomas, especially in those with clinical presentation outside lymphatic nodes. Undoubtedly, the accumulated experience from CAR T-cell treatment of leukemia and lymphoma has provided crucial knowledge about some key factors (both tumor and T-cell related) essential for the advancement of immunotherapy in other types of tumors.

In this review we summarize key predictors of CAR T-cell efficacy in lymphomas and outline mechanisms of immune escape related to both solid tumors and lymphomas in order to identify the most promising trends for future development of CAR T-cell therapy.

## 2. CAR T-Cell Therapy

**Figure 1:**
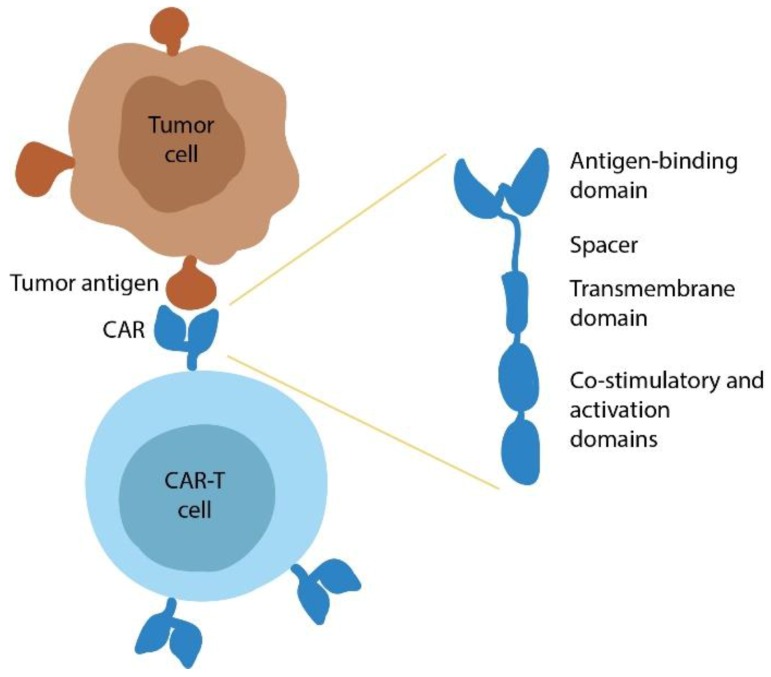
Schematic representation of a chimeric antigen receptor (CAR) T-cell and its interaction with the tumor cell. The CAR contains two primary functional components: an antigen-binding domain (derived from variable region of the monoclonal antibody to an antigen) and an intracellular activation domain (derived from immunoreceptor tyrosine-based activation motifs (ITAMs) of CD3ζ and often also including one or more co-stimulatory domains, e.g., CD28, 4-1BB) for signal transduction. Antigen-binding and transmembrane domains are connected via a flexible spacer that partially contributes to the efficiency of target recognition [8,9].

CAR T-cells are genetically modified T-cells expressing chimeric-antigen receptor that enables them to specifically recognize and bind the target tumor antigen (e.g., CD19) followed by cytotoxic elimination of the tumor cells via perforin/granzyme-induced apoptosis (Figure 1). CARs are transmembrane receptor proteins consisting of several functional domains. This includes an extracellular single-chain variable fragment (scFv) derived from the antigen-recognizing component of an antibody, a hinge/spacer sequence, a transmembrane domain, and an intracellular domain for signal transduction.

The progressive development of CAR technologies is often categorized into sequential generations of which the fourth generation is now considered to be the most advanced. The term “generation” was initially used to describe the domain architecture of CARs but now it commonly refers to CAR-T cells themselves. The first-generation CARs contain scFvs, transmembrane domain, and intracellular CD3ζ immunoreceptor tyrosine-based activation motifs (ITAMs). The second-generation CARs carry an auxiliary intracellular co-stimulatory domain, such as CD28, CD137, and several others. The most prominent examples of the second-generation CAR T-cell product are Kymriah^®^ and Yescarta^®^, approved by the FDA in 2017. The third-generation CARs include two or more additional co-stimulatory domains. The fourth-generation CAR T-cells additionally express various co-stimulatory components such as cytokines, antibodies, or other functional proteins.

## 3. Solid Tumors Are Prominently Heterogeneous—One Approach Does Not Fit All

Historically, tumors are classified according to parameters such as histology, tissue, and organ of location. Today the analysis of immunohistochemical patterns has become essential for tumor specification. Some histological tumors, such as melanoma and certain subsets of colon and lung cancer, are known for their high immunogenicity and good response to treatment with checkpoint inhibitors (CIs). For example, ~40% of patients with metastatic melanoma achieved over 4 years progression-free survival (PFS) upon treatment with a combination of ipilimumab (anti-CTLA4) and nivolumab (anti-PD-L1), whereas in ovarian and pancreatic cancers such treatment demonstrated modest to no effect [10]. The survival achieved in these patients was truly outstanding; this cohort would have been otherwise incurable in the pre-CI era. On the other hand, certain melanoma subsets remain resistant to CI while in other types of tumors a positive effect has occasionally been observed (e.g., long-lasting CR in one patient with resistant ovarian cancer treated with nivolumab [11]). Accordingly, some novel tumor classifications were proposed for better prediction of a potential response to immunotherapy in a given patient in order to prescribe individual treatment. In 2017 the FDA approved anti-programmed death-ligand 1 (PD-L1) immunotherapy with pembrolizumab for a subset of tumors with microsatellite instability resistant to conventional treatment [12]. This was the first example of therapy prescription without strict compliance to histopathological analysis.

There are multiple other strategies for prediction of the response to programmed cell death protein 1 (PD-1) immunotherapy. One of the most logical seems to be the assessment of PD-L1 expression on tumor cells and tumor microenvironment using immunohistochemical analysis. The FDA has approved PD-L1 expression analysis as a diagnostic marker for anti-PD-1 therapy in patients with non-small-cell lung carcinoma (NSCLC), and pembrolizumab is an example of a drug approved for patients with PD-L1-expressing NSCLC. Unfortunately, this diagnostic approach had moderate accuracy and cases of responders with low PD-L1 expression and non-responders with high PD-L1 expression were reported. Supporting this, multiple studies could not confirm an association between PD-L1 expression and response to CI treatment [13]. The discrepancy of opinions on the effectiveness of such approach may be explained by utilization of different assessment protocols, including variations in cutoff levels, percentage of positive cells versus staining intensity, PD-L1 expression on tumor cells versus microenvironment. An alternative approach, termed “Immunoscope”, is based on standardized histological evaluation of CD8^+^ T-cells in tumor parenchyma and margins [14,15]. Additionally, the list of potential predictors includes evaluation of tumor mutational burden (TMB) [16], combined assessment of PD-L1 expression and presence of tumor-infiltrating lymphocytes (TIL) [17], transcriptome analysis for detection of “inflammatory” genetic signatures [18], and the assessment of neoantigen load and immunogenicity [19,20]. In general, combinational approaches based on simultaneous assessment of multiple parameters could potentially demonstrate better predictive ability. Accordingly, tumors with modest PD-L1 expression but significant TMB or high neoantigen burden might be an appropriate target for CI therapy.

Other than CIs, we know very little about the prediction of therapeutic efficacy for CAR T-cells. Multiple reviews describe barriers to successful clinical application of CAR T-cell therapy, other T-cell-based therapies, and CIs [21,22,23]. The key mechanisms of tumor immunoevasion include: (1) impaired molecular trafficking into the tumor (caused by pro-tumoral endothelium [24,25], altered chemokine profile [26,27,28], and stiff stroma [29]); (2) microenvironment-mediated alterations that include hyperacidity and increased potassium level [30], presence of myeloid derived suppressor cells (MDSCs) [31,32], tumor-associated macrophages (TAMs) [33], and their cytokines/chemokines; and (3) modulation of tumor-specific molecular pathways [34], e.g., phosphatase and tensin homolog (PTEN)/phosphoinositide 3-kinase (PI3K) [35,36,37], Janus kinase 1 (JAK1)/2 [38,39,40], isocitrate dehydrogenase (IDH) [41,42], interferon-gamma (IFNγ) and tumor necrosis factor alpha (TNFα) [43], and microRNAs and exosomes [44].

Apparently, tumors engage various defensive mechanisms in different situations, as highlighted by Chen and colleagues [29]. They divided tumors into three categories: (1) “inflamed”, with enriched T-cells located mostly in tumor parenchyma; (2) “immune-excluded”, with enriched T-cells located within dense tumor stroma that prevents T-cell trafficking into parenchyma even upon CI therapy; and (3) “immune desert” tumors lacking T-cells. Bioinformatics analysis of transcriptome data from The Cancer Genome Atlas allowed Thorsson et al. to cluster non-hematological tumor specimens into six signature categories [41]. Two signatures (IFNγ-enriched and inflammatory) showed significantly improved clinical outcome regardless of the treatment and thus predicted prognosis without strict consistency with tumor histopathology. In addition, another signature demonstrated the prominent role of a transforming growth factor beta (TGFβ)-dependent tumor escape mechanism. Despite the overall orderliness, the major disadvantage of this classification system compared to histopathological analysis is its inability to spatially locate immune cells, including T-cells, across the tumor. Therefore, tumors with typical T-cell exclusion phenotype could potentially fall into IFNγ or inflammatory signature categories due to merely increased number of T-cells. This ambiguity might be the reason why some tumors, such as stroma-rich pancreatic cancer, demonstrate inflammatory/IFNγ-enriched properties in up to 50% cases, and yet still have some of the worst clinical prognoses even upon CI treatment [41,45].

### Is Selection of an Optimal Target a Cornerstone of CAR T-Cell Therapy?

The scarcity of appropriate targets commonly heads the list of obstacles towards successful clinical application of CAR T-cell therapy against solid tumors (Table 1). Some approaches for generating tumor- and patient-specific CAR-Ts were proposed using lymphoma as a model; however, manufacturing of the cell product requires several weeks and is not fully applicable for solid tumors [46].

The variety of antigens amenable for CAR-T immunotherapy is limited due to the overall tumor heterogeneity and nonuniformity of antigen expression [55]. The suitable antigens are often shared between the tumor and healthy tissues differing only in antigen density that makes potential toxicity a major issue. With regards to solid tumors there is no healthy tissue that could be harmlessly sacrificed, as opposed to CD19 targeting of lymphoma vs. healthy B-cells.

At the same time, tumor-exclusive antigens actually exist but are relatively rare. One of them is tumor-specific epidermal growth factor receptor variant III (EGFRvIII) [56] overexpressed by glioblastoma in ~30% of newly diagnosed cases [57]. As expected, following CAR T-cell therapy glioblastoma tends to decrease antigen expression typically to ~50% of initial levels in those cases where CAR-T persistence is achieved [52]. In this study, the antigen expression was completely lost only in two of seven evaluated cases. However, low response rates (only one in 10 patients had an 18-month ongoing response) suggest that although selection of target antigen is highly important it is not the cornerstone issue for immunotherapy of glioblastoma and solid tumors in general. We further speculate on the optimal selection of target antigens in a section devoted to future perspectives of the therapy.

## 4. What Do We Know about Lymphoma?

At first glance hematological malignancies have a set of unique features making them a favorable target for immunotherapy: (1) predominant localization at common sites of lymphocyte persistence, such as lymph nodes and bone marrow that facilitate CAR T-cells trafficking; (2) uniformly expressed lineage-specific antigens wherein antigen-bearing healthy cells could be safely sacrificed (e.g., B-cell malignancies); and (3) physical barriers, such as fibrotic stroma and immunosuppressive microenvironment that are significantly less prominent compared to solid tumors [58]. However, each of these characteristics is somewhat controversial and does not fully define the scope of CAR T-cell therapy, e.g., positive response was reported for the treatment of lymphoma in central nervous system (CNS) [49] and skin relapse [59]. This suggests that knowing localization of the tumor within the lymph nodes is not required for successful immunotherapy.

### 4.1. Antigen Selection: Is CD19 the Ideal Target?

CD19 antigen appears to be a highly attractive target for CAR-T therapy due to several main reasons: (1) it is ubiquitously expressed on tumor cells; and (2) apparent damage to patient’s adaptive immunity upon loss of B-cells could be successfully overcome with immunoglobulin replacement therapy.

Nevertheless, antigen loss is frequently reported in ALL [60] and less frequently in NHL [61,62,63]. Abramson et al. conclude that although CD19 is heterogeneously expressed in lymphoma cells, the incidence of CD19 relapse in diffuse large B-cell lymphoma (DLBCL) is only approximately 10% [64]. The clinical data from ZUMA-1 trial supports these findings as CD19 loss was confirmed only in three out of 11 relapsed patients [63]. Interestingly, leukemic cells lacking extracellular CD19 domain can be traced at the time of initial diagnosis before any therapy is administered thus implying that some ALLs and lymphomas are heterogeneous enough to resist CAR T-cell therapy [65]. Hence, tumors do not always follow the evasion strategy based on antigen loss despite the implicit presence of such clones within the tumor. It should also be mentioned that CD19-deficient/mutated B-cells may have lower proliferative potential due to the necessary presence of CD19 for B-cell development and survival [66,67]. The above-mentioned cases of ALL CD19 relapses are associated with expression of preserved intracellular and truncated extracellular CD19 domains that nonetheless enable functional signaling. However, there are also other targets in hematology (Table 2), such as B-cell maturation antigen (BCMA), for which CAR-T treatment resulted in near 100% objective response with high CR rate, yet it was not curative [1,68]. A successful example includes anti-CD22 CAR T-cell therapy (*n* = 52) that resulted in high remission rates in ALL patients but was followed by 64% relapse [69]. Importantly, in this case 57.5% of the patients received prior anti-CD19 CAR-T treatment that partly explains high relapse rates. In contrast to CD19 the functions of CD22 antigen in B-cells are somewhat controversial as this molecule exerts both inhibitory and stimulatory influence on B-cell proliferation [70]. Compared to normal cells the proliferation of CD22-deficient cells was slightly reduced in response to IgM stimulation and increased in response to lipopolysaccharide [71]. Thus, although the presence of CD22 is not strictly crucial for B-cell development and function, it still represents an example of the appropriate target for CAR T-cell therapy.

### 4.2. Immunosuppressive Microenvironment Also Matters in Lymphoma

Solid tumors are often accompanied by a potent microenvironment protecting them against the immune system. Similarly, some lymphomas appear to essentially depend on tumor microenvironment (TME) (e.g., follicular and Hodgkin’s lymphomas) that enables recruitment and modulation of pro-tumor non-malignant cells. Only few subtypes among the most aggressive lymphoid tumors do not require intensive microenvironment to support their proliferation (e.g., Burkitt lymphoma) [75]. Specific chemokine receptors and adhesion molecules make lymphoma cells capable of “homing in” on certain body sites (e.g., lymph nodes for CCR7 and CXCR4/CXCR5 [75]) and this property might be the basis of primary disseminated clinical presentation of lymphoma.

“Solid tumor-like” participators of the microenvironment landscape, such as cytokines (IL4, IL6, IL10, and TGFβ) and cells (M2 macrophages, N2 neutrophils, MDSCs, Tregs) are often found across lymphoma tissues [76,77]. In contrast to solid tumors we still know very little about angiogenesis in lymphomas [75], however similarities with solid tumors exist and vascular endothelial growth factor (VEGF) is clearly a player is that process [78]. However, analogous to some trials in solid tumors [79] targeting the VEGF pathway in DLBCL by a combination of bevacizumab with R-CHOP (a chemotherapy regimen used for the treatment of NHL) did not significantly improve outcomes but instead increased toxicity [75]. Lymphoma cells express CD70, CD80, CD86, and PD-L1 immune checkpoints that downregulate effector T-cells including Th17 [80], and that allowed clinical approval of CIs for Hodgkin’s lymphoma (HL) and primary mediastinal B-cell lymphoma [81,82]. Cancer-associated fibroblasts (CAFs) promote tumorigenic features of the microenvironment and are often studied merely within the context of bone marrow, as part of multiple myeloma and hairy cell leukemia [83].

Although the composition of lymphoma extracellular matrix (ECM) is relatively well known [78], abundant stiff ECM seems to be underrepresented within lymphoma TME, especially in comparison to stroma-rich tumors, such as pancreatic cancer [84]. Vasculature within lymphoma is known to have the same thickness as within the normal lymphoid tissue suggesting the lack of perivascular fibrosis that facilitates lymphocyte trafficking [85]. A recent study identified a decrease of collagen-modifying enzymes in malignant lymphoid tissues implying a lower frequency of lymphoid stromal cells (e.g., fibroblastic reticular cells and follicular dendritic cells) within the tumor [86]. As far as we know, the only type of lymphoma rich with stiff ECM is the nodular sclerosis subtype of HL, which however does not have a significantly diminished treatment susceptibility [87].

### 4.3. T-Cell Composition Is Critical Even in Immunodeficient Mouse Models

FDA-approved anti-CD19 Yescarta^®^ and Kymriah^®^ are produced from the whole population of peripheral blood mononuclear cells (PBMCs) without separation of specific T-cell subtypes [88,89]. Today it is well understood that subtype composition of CAR T-cell product is an essential parameter that defines its key therapeutic features such as safety and efficacy [54,90,91,92,93,94]. This optimal subtype composition was argued since the first CAR-T clinical trial in neuroblastoma that identified increased percentage of CD4^+^ T-cells and CD45RO^+^CD62L^+^ central memory T-cells (TCM) in the infused product as a predictor of prolonged persistence [54]. Juno Therapeutics Inc. is currently developing JCAR014 and JCAR017 products with defined 1:1 composition of CD4^+^ and CD8^+^ TCMs [92,93] that has demonstrated substantially lower side effects [73,92]. Schmueck-Henneresse et al. showed that CAR T-cells derived from naïve T-cells had decreased proliferation after CAR-specific activation compared to other subtypes [95]. Moreover, the same study reported that naïve T-cells vigorously proliferated during ex vivo expansion and eventually accounted for up to 89% of the resulting CAR T-cell population. Fraietta et al. applied Kymriah^®^ CAR T-cells clinically manufactured for patients with chronic lymphocytic leukemia (CLL) to treat NOD-SCID/γc^−/−^ (NSG) mice engrafted with NALM-6 leukemia cells [96]. They found that mouse survival heavily depended on whether CAR-Ts were generated from responder or non-responder patients, and that the percentage of memory stem T-cells (TSCMs) was significantly higher in responders. The authors identified potential predictors of successful CAR T-cell therapy, such as the presence of CD27^+^CD45RO^–^CD8^+^ T-cells (related to long-lived memory cells) in premanufacturing leukapheresis product and the prevalence of CD27^+^PD-1^–^CD8^+^ CAR T-cells, and also activation of memory-related genes involved in IL6/STAT3 signaling pathway. Importantly, CAR T-cell intrinsic fitness was found to be a critical factor for the treatment efficacy even in immunodeficient mice partly lacking TME.

Rossi et al. assessed the polyfunctionality (secretion of more than 2 cytokines by a single cell) of CAR T-cells on a cohort of 22 patients suffering from NHL and revealed the ability of lymphocytes to simultaneously secrete inflammatory IL17A and effector IFNγ [97]. Approximately 20–25% of all cytokine-producing CAR T-cells were found to be polyfunctional and associated with higher rates of successful therapy. Moreover, IL17A-producing polyfunctional CD4^+^ CAR T-cells were also found to contribute to a positive treatment response, possibly by sharing Th17 cell properties and reprogramming TME. The authors did not identify any difference between CAR T-cell phenotype in responders and non-responders by conventional flow cytometry. Some have suggested that tumors enriched with Th17 signature have more favorable clinical outcomes [41], and that Th17 cells are generally associated with improved cancer survival [98]. Interestingly, Neelapu et al. report no statistical association between CD4^+^:CD8^+^ subpopulations ratio and clinical efficacy or toxicity for Yescarta^®^ [63]. It is important to note that these cells and the ones investigated by Rossi et al. [97] are both CD3ζ/CD28 CAR-Ts, suggesting that for some CAR T-cell products subtype composition might be less critical.

### 4.4. Treg Subsets May Be Overrepresented in Peripheral Blood of Patients with Solid Tumors and May Influence CAR T-Cell Composition

An increased proportion and absolute population of CD4^+^CD25^+^CD45RA^−^ T-cells (Tregs) were revealed in 42 patients with endothelial cancer [99]. The median percentage of Tregs accounted for 12.5% and was elevated to 33.5% for a single stage IV lung cancer case. Patients received neither immunosuppressive therapy nor chemotherapy for at least 3 months; thus, the percentages seem to reflect their constant immune status. In contrast to this, a recent study has compared proportions of suppressor cell subpopulations in lymphoma patients (*n* = 43) and healthy donors [100]. No significant difference in Treg populations between the groups was observed, although some non-T-cell populations (MDSC and NK regulatory cells) were elevated in the patients. The above-studied cohorts were similar (Europeans, ~60 years), however direct comparison of the results cannot be totally correct due to variations in applied methodology: (1) calculation of Treg percentages in total population of PBMCs [100] or blood cells [99]; and (2) different definition of Treg phenotypes. Some studies on lymphoma patients have similarly found no variation in Treg levels in comparison to healthy volunteers [101,102], while others reported several-fold difference regardless of nationality, age and disease status (newly diagnosed vs. relapsed) [102,103,104].

Nevertheless, numerous other studies found increased Treg blood levels of up to 19% of total CD4^+^ population in gastric cancer and 27% in esophageal cancer patients in comparison to only 9% in healthy donors [105,106,107,108,109,110]. Chellappa et al. reported an increase of total Treg population in pancreatic cancer patients that was exclusively due to the expansion of FOXP3^+^RORγt^+^ subset possessing both inflammatory and immunosuppressive properties [110].

Currently, there is limited information on the assessment of Treg population in CAR T-cells. However, it would be reasonable to expect that increased Treg levels in patients with solid tumors could affect final composition and expansion of CAR T-cell product. Some strategies for overcoming Treg proliferation within CAR-T product and in patient body after infusion are reviewed in [111].

### 4.5. Clinically Reported Predictors of CAR T-Cell Efficacy in Lymphoma and Leukemia

**Figure 2:**
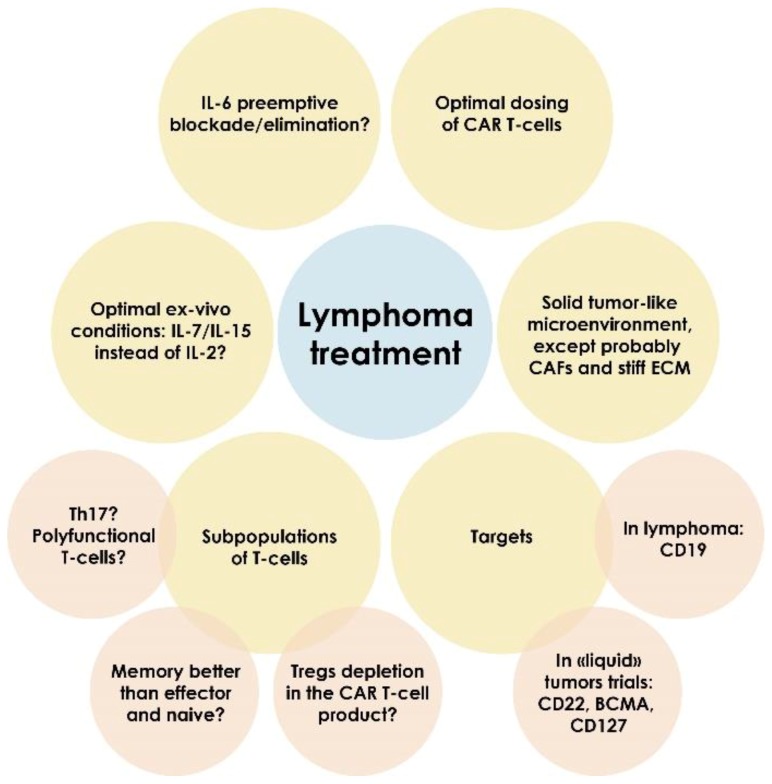
The highlights of experience from treatment of lymphoma with CAR T-cells. Clinical administration of anti-CD19 CAR T-cells resulted in accumulation of vast experience specifying potential predictors of therapeutic response. Some of them are presented in this figure. ECM—extracellular matrix.

Large clinical trials studying cohorts of patients suffering from different hematological diseases (e.g., CLL, NHL and ALL) contributed to the broader understanding of CAR T-cell therapy efficacy and the role of various signaling pathways in this process. For example, IL15 in serum positively correlates with CAR T-cell peripheral levels [91,112], therapy efficacy [112] and severe neurotoxicity [91,112]. IL6 is associated with substantial toxicity and reduced clinical benefit from immunotherapy thus preemptive elimination of this cytokine from circulation using antibodies or by other means seems reasonable [113]. However, some recent studies report the secretion of IL6 by CAR T-cells [96,114] and the importance of the IL6/STAT3 signaling pathway for the treatment efficacy [96]. Juno Therapeutics Inc. demonstrated correlation of peak CAR T-cell serum levels with both efficacy and toxicity of the therapy [90,113]. They developed a logistic regression model that potentially allows for selection of an optimal therapeutic window. Finally, lymphodepletion, i.e., chemotherapeutic in vivo elimination of patient T-cells, was found to be crucial, as anti-CAR immune response was observed when anti-CD19 CAR T-cells were infused without prior lymphodepletion [115,116,117]. Moreover, combination of lymphodepletion with cyclophosphamide and fludarabine resulted in better CAR T-cell expansion [92,118], higher serum CAR-T peak [119,120], but increased toxicity [90,119]. The recent data from the American Society of Clinical Oncology support these observations [47,48,49,50,51], as higher treatment efficacy was achieved with pre-infusion lymphodepletion. The factors significant for CAR T-cell therapy efficacy are summarized in Figure 2.

## 5. Future Perspectives for CAR T-Cell Therapy against Solid Tumors

### 5.1. Optimization of T-Cell Composition and Fitness

We have discussed the importance of T-cell composition in patients suffering from lymphoma/solid tumor and CAR T-cell product use. Importantly, it might be possible to indirectly regulate the T-cell composition by using optimized expansion protocols. Schmueck-Henneresse et al. reported that a combination of IL7 and IL15 increased the yield of CAR T-cells derived from TSCMs and TCMs in comparison to IL2 that is widely used for proliferation of clinical grade CAR-Ts, including Yescarta^®^ and Kymriah^®^ [95]. Furthermore, recent studies by Vodnala et al. revealed that TIL dysfunction caused by caloric restriction and increased K^+^ concentration within the tumor leads to decreased T-cell activation and differentiation [30]. The authors adopted this tumor-mediated mechanism to demonstrate that T-cells that were ex vivo preconditioned at high K^+^ concentration subsequently in vivo maintained a less differentiated phenotype. These T-cells also achieved better persistence within tumors than unconditioned cells resulting in prolonged survival and better tumor regression in a melanoma mouse model. Other strategies to limit the cell differentiation include pharmacological blockade of phosphoinositide 3-kinase (PI3K) or shortening the duration of ex vivo expansion [121].

### 5.2. Design of CAR T-Cell Products

The idea of dual antigen targeting, especially in solid tumors, has long been envisaged as a highly promising and fruitful therapeutic approach. Hegde et al. addressed the question of the optimal number of simultaneously targeted antigens and studied the expression of human epidermal growth factor receptor 2 (HER2), IL13Rα2, and EphA2 antigens in tumor samples obtained from glioblastoma patients [122]. The authors confirmed that mitigation of tumor antigen escape via tandem targeting of HER2 and IL13Rα2 antigens with autologous CAR T-cells resulted in a clear positive impact on tumor elimination both in vitro and in vivo, whereas addition of the third antigen did not yield any significant beneficial effect. Moreover, they confirmed that bi-specific CAR T-cells simultaneously expressing both anti-HER2 and anti-IL13Rα2 CARs provide more sustained activity than pooled cells with individually expressed CARs. The authors further report a tandem CAR exodomain TanCAR with combined structural features of both HER2-binding and IL13Rα2-binding domains [123]. These TanCAR cells resulted in substantially higher cytokine release and intensity of tumor cell lysis as well as prolonged mouse survival, in comparison to the abovementioned pooled and bi-specific CAR T-cells. Interestingly, expression of T-cell exhaustion markers (PD-1, lymphocyte activation gene 3 (LAG3), and T-cell receptor (TCR) inhibitory molecule 3 (TIM3)) on TanCAR cells did not substantially differ from bi-specific CAR-Ts that may result in their higher clinical potential.

The question of primary importance is the optimal design of CAR itself and the enhancement of immunological synapse between the CAR and the tumor-specific target antigen. For example, scFv affinity, hinge and spacer length may influence CAR T-cell activity [124], as further discussed below.

Moreover, the concept of T-cells with synthetic Notch (synNotch) receptors introduced by Roybal et al. demonstrated transformation of T-cells into “soldiers” with fully customized response to target antigens [125]. This includes cytokine release, T-cell differentiation or local delivery of non-native therapeutic payloads, such as antibodies.

### 5.3. Promising Combinational Approaches

Typically, the tumor engages a unique combination of driving mechanisms that include TGFβ-dependent T-cell exhaustion by a range of immune checkpoints and recruitment of local microenvironment, e.g., tumor-associated M2 macrophages. In this situation, a promising solution would be administration of CAR T-cells resistant to tumor-specific impact or their combinations with other therapies. Certain modifications could enhance anti-tumor armory of CAR T-cells, for instance, expression of dominant-negative receptors that block inhibitory signaling within T-cells, e.g., TGFβ [126] or PD-1 [127]. A phase I clinical trial of prostate-specific membrane antigen (PSMA)-based CAR T-cells with co-expression of dominant-negative TGFβ receptor II (TGFβRII) in patients with R/R metastatic prostate cancer is ongoing (NCT03089203). Other advanced CAR-T strategies include release of auxiliary co-stimulation molecules [128] and cytokines [129]. A more flexible option is to combine CAR T-cells with a range of CIs, antibodies or kinase inhibitors. Unfortunately, to date TME-modifying drugs are represented only by a handful of clinically available CIs, e.g., anti-PD-1/PD-L1 (nivolamab, pembrolizumab, cemiplimab, atezolizumab) and anti-CTLA4 (ipilimumab) drugs. A range of novel CIs, such as anti-LAG3 (IMP321/Immuntep^®^), are being investigated in clinical trials at Phase I or II [22]. Another approach based on combination of anti-PD-1 and anti-TGFβ therapy is being tested for the treatment of advanced-stage lung cancer/hepatocellular carcinoma (NCT02423343) and metastatic pancreatic cancer (NCT02734160) [130]. Plerixafor, anti-TAM drug, resulted in near complete disappearance of malignant cells in mouse model of pancreatic cancer [33] and is currently being evaluated in patients with pancreatic, ovarian and colorectal adenocarcinomas (NCT02179970, NCT03277209). Finally, abovementioned synNotch T-cells can be administered in combination with conventional T-cells in order to modulate tumor microenvironment [125]. We should stress that clinical validation of novel CAR-T therapies is critical as intercellular interactions between the tumor and the fully functional human immune system are not always reproduced in immunocompromised animal models. Indeed, attempts to inhibit VEGF pathway were undertaken in pre-clinical settings by using a combination of anti-VEGF bevacizumab and anti-GD2 CAR T-cell therapy that led to a prolonged survival of SCID/Beige mice (orthotropic xenograft model of human neuroblastoma) [131]. However, combination of bevacizumab with the standard of care lomustine, albeit without immunotherapy, demonstrated no improvement in survival of R/R glioblastoma patients [79]. Similarly, no positive outcomes were achieved for combination of bevacizumab with chemotherapy in lymphoma patients [75]. Other preclinically successful therapies, such as IL12 expressing TRUCK [132], upon clinical translation showed unexpected toxicity and lack of therapeutic effect (NCT01236573, NCT01457131), eventually resulting in termination of clinical trials.

### 5.4. Universal CAR T-Cells

Limited clinical availability of CAR-T therapy is due to high costs, time-consuming manufacturing and production failures that together make the concept of universal CAR-Ts (UCAR-Ts) particularly attractive. In 2017 Qasim et al. reported two infants with relapsed ALL treated with anti-CD19 UCAR-Ts genetically engineered with transcription activator-like effector nuclease (TALEN) to disrupt expression of TCRα chain (protection from graft-versus-host disease, GvHD) and CD52 (to prevent rejection) [133]. The children remained in CR after 12 and 18 months post-treatment. Similarly designed anti-CD123 UCAR-Ts are currently being investigated in a Phase I clinical trial in patients with refractory AML [134].

A different approach was utilized by Celyad Inc. to develop two CAR T-cell products (allogeneic CYAD-101 and autologous CYAD-01) bearing natural killer receptors (NKRs) targeting a range of ligands expressed across various cancers. The important feature of CYAD-101 is the expression of the inhibitory peptide T-cell receptor (TCR) inhibitory molecule (TIM) that lowers the TCR-signaling and thus the probability of developing GvHD. These products are now being investigated in Phase I clinical trials. In the alloSHRINK trial (colorectal cancer, CYAD-101) two out of 12 patients achieved partial remission (PR) and another five achieved SD [135]. In THINK trial (AML, CYAD-01) anti-leukemic activity was achieved in six out of 13 patients [136]. No significant toxicity was reported for either of the CAR T-cell products.

Allogeneic CAR T-cell therapy may face significant obstacles on its way to successful clinical application. The major challenge is to achieve prolonged CAR T-cell persistence without continuing depletion of patient immune cells described by Qasim et al. [133]. The approach of disrupting human leukocyte antigen (HLA) expression in CAR T-cells is substantially limited by NK- and NKT-cells that are likely to eliminate HLA-negative cells. Additional modifications in HLA-H, HLA-E, or HLA-G expression are required to keep therapeutic cells protected from the host NKs.

A similar concept of the bank for “universal” stem cells for the purpose of artificial solid organ transplantation was addressed by Taylor et al., who bioinformatically confirmed that a selection of 150 homozygous HLA-typed volunteers covers 93% of the UK population with a fully matched donor tissue [137]. In theory, selective depletion of HLA-A and HLA-B while preserving HLA-C on CAR T-cells could potentially limit the essential pool of allogeneic “off-the-shelf” cell products to several dozens or even less. Nevertheless, allogeneic cells with retained expression of either HLA class I or II still hold a significant threat of immunogenicity in a patient due to minor histocompatibility antigens (MiHAgs) [138] derived from near-infinite diversity of single nucleotide polymorphisms (SNPs) that vary in the donor and a patient [139]. These antigens contribute to GvHD (in hematopoietic stem cell transplantation) or transplant rejection (in solid organ recipients) and could also limit the persistence of allogeneic CAR T-cells.

## 6. Can T-Cells with Conventional TCRs Still Play Any Role in Cancer Treatment? TCR Versus CAR?

**Figure 3:**
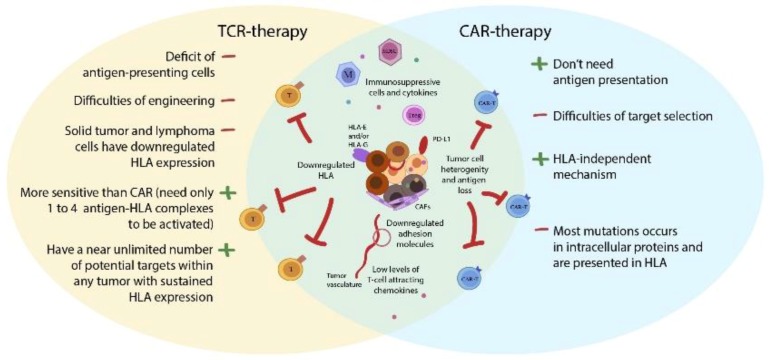
Advantages and disadvantages of CAR T-cell therapy in comparison to TCR therapy. CAR T-cells possess certain advantages over TCR therapy, such as independence from HLA and DCs/APCs. Still, the TCR-based approach has an important benefit based on countless various antigen peptides presented by HLA, thus resulting in a multitude of potential targets. Abbreviations: CAR-T—T-cells with CAR, T—T-cells with TCR, M—macrophage, MDSC—myeloid derived-suppressor cell, Treg—regulatory T-cell, CAF—cancer-associated fibroblasts, PD-L1—programmed death-ligand 1, HLA—human leukocyte antigen; DCs—dendritic cells; APC—antigen-presenting cell.

The primary feature of CAR T-cell is its HLA-independent mechanism of action, yet CAR does not provide a panacea because the number of the tumor-specific and tumor-associated antigens is limited. Paradoxically, the limitation of TCR-based therapy is HLA-dependence, though balanced by almost infinite number of potential target antigens (Figure 3). Mutations typically occur in intracellular proteins and then the multitude of relevant antigen peptides is presented by HLA [58], making such tumor cells not directly amenable for targeting by CAR T-cells. In support of this, a reportedly high immunogenicity of p53-mutated tumors can be explained by HLA-mediated presentation of mutant p53-derived neoantigens [140]. The ubiquity of oncogenic p53 mutations across different types of human cancers results in poor prognosis and underlines the potential clinical utility of TCR-mediated p53 targeting. Laumont et al. analyzed the abundance of neoantigens derived from non-coding regions of human genome/alternative reading frames (RFs) and predicted that their number is much higher than that of the antigens derived from canonical RFs of known protein-coding regions [141]. Protein-coding regions represent only ~2% of the human genome, whereas transcriptome covers about ~75% and includes “non-protein-coding” transcripts that are nonetheless translated [142,143]. Considering that 99% of genetic mutations are located in those allegedly non-coding regions the number of potential neoantigens is expected to be high even in tumors with low TMB.

Harris et al. performed direct in vitro comparison of TCR- and CAR-based therapeutic approaches by engineering two single-chain TCRs against melanoma antigen recognized by T-cells 1 (MART1)/HLA-A2 or Wilms tumor protein (WT1)/HLA-A2 and respective CARs that recognize these antigen/HLA complexes [114]. Despite higher surface expression, CARs (CD3ζ or CD3ζ/CD28) were still 10 to 100-fold less sensitive to their matching peptide/HLA-A2 complexes than TCRs. These findings lead to speculation about potential using CAR T-cells for targeting antigens with high expression in tumors and low in healthy tissues. However, a case report of fatal toxicity, caused by CAR T-cells action against healthy tissues with low antigen expression, suggests that CARs have to be designed extremely carefully to avoid disproportionate sensitivity issues [144]. Parameters to be considered include the nature of the recognition domain (either TCR-derived or antibody-derived) and regulation of its affinity to the antigen [124], as well as the length of non-signaling spacer [8,9]. Taking into account relative surface density of the tumor antigens, CAR-Ts appear to have a higher activation threshold (~200 antigens per tumor cell) than T-cells (1–4 antigen-HLA complexes per tumor cell) [124]. The smart solution to the potential toxicity problem involves suicide genes that can be controllably activated in vivo and result in safe elimination of CAR-T cells inside the patient [145]. Presumably, CAR T-cells could be administered in combination with conventional T-cells and NKR T-cells for simultaneous dual/triple targeting of HLA-positive and HLA-negative cells in order to completely exterminate the tumor.

## 7. Conclusions

CAR T-cell therapy holds a great promise for the treatment of hematological malignancies. The extensive experience from lymphoma clinical studies provides us with the understanding that, although selection of the surface antigen is a primary issue to be tackled, it is not the only one. For solid tumors the challenge is the need to overcome the local microenvironment in terms of its immunosuppressive capacity and physical barriers, e.g., ECM that is probably more pronounced in solid tumors than in lymphomas [146]. Another significant factor to consider is the choice of a particular T-cell subset for generating CAR T-cells. Considerable heterogeneity of tumor antigens and the presence of certain antigens on both tumor and healthy tissues could be addressed by T-cells with dual/multiple CARs as a way of improving efficacy and decreasing toxicity. Moreover, the key to unleashing the immense potential of CAR T-cells might be in combination with other treatment modalities, for example with CI therapy. This approach could be highly efficient in patients with immunologically “hot” tumors while others might require a different avenue by targeting ECM and T-lymphocyte exclusion pathways, e.g., VEGF or TGFβ. A number of new medications targeting TME are expected to become clinically available in the foreseeable future, thus adding to the growing list of personalized combinational therapies. Transcriptome profiling technologies could assist in predicting the potential success of new treatments based on genetic signatures (e.g., TGFβ signature for anti-TGFβ drug administration). Several companies, such as the BostonGene Inc., Foundation Medicine Inc., and NanoString Inc. are developing novel approaches for deeper personalization of immune therapies. We can envisage that the future for treatment of solid tumor relapses might be in adoptive immune therapy combined with a range of targeted “enhancers” that operate in accordance with the specific TME and TMB profiles in a given patient.

## Figures and Tables

**Table 1:** Recent achievements in the field of CAR T-cell therapy against solid tumors. CNS—central nervous system; CR—complete response; PSCA—prostate stem cell antigen; MPM—malignant pleural mesothelioma; PR—partial remission; SD—stable disease; LD—lymphodepletion; PD—progressive disease; PD-1—programmed cell death protein 1; OS—overall survival; TCR—T-cell receptor; EGFRvIII—epidermal growth factor receptor variant III; HER2—human epidermal growth factor receptor 2; GD2—disialoganglioside.

Target	Cancer Type	Number of Patients	Results	Clinical Trial or Reference	Comments
Mesothelin	MPM (mesothelioma)	14	2 CR (62 and 39 weeks ongoing); 5 PR and 4 SD	NCT02414269 [47]	Regional delivery LD anti-PD-1
PSCA	Metastatic pancreatic, gastric, or prostate cancers	15	8 SD and 3 PD	NCT02744287 [48]	PSCA-CD3ζ CAR and a rimiducid (Rim)-inducible MyD88/CD40 co-activation switch Different LD
CD19	CNS lymphoma	9	4 CR	NCT02631044 [49]	Lisocabtagene maraleucel LD
	Synovial sarcoma	9	3 PR	[50]	TCR LD
Claudin 18.2	Gastric and pancreatic	12	1 CR, 3 PR, 5 SD	NCT03159819 [51]	LD
EGFRvIII	Glioblastoma	10	OS ≈ 8 months 1 CR (18 months)	[52]	Combination with surgery when clinically indicated
HER2	Sarcoma	17	4 SD	[53]	No LD
GD2	Neuroblastoma	11	3 CR, of them—2 prolonged CR	[54]	No LD

**Table 2:** CAR T-cell therapies for the treatment of hematological cancers. ALL—acute lymphoblastic leukemia; AML—acute myeloid leukemia; BCMA—B-cell maturation antigen; MM—multiple myeloma; NHL—В-cell non-Hodgkin’s lymphoma; PFS—progression-free survival.

Target	Cancer Type	Number of Patients	Results	Clinical Trial or Reference	Comments
BCMA	MM	43	12 months median PFS 91–100% ORR 40% CR	[1,72]	LD
CD123	AML	24, 12 infused	3 CR 1 morphologic CR 1 PR 2 SD	NCT02159495 [7]	LD
CD22	ALL lymphoma	52	CR 72% 6 months median PFS 64% relapse	[69]	LD 58% had prior CD19 CAR-T
CD19	NHL	91	12 months OS 63% 6 months CR 50%	TRANSCEND NHL 001 [73]	LD Defined CAR-T composition
CD19	NHL	101	18 months OS 52% CR (15.4 months) 40%	ZUMA-1 [63]	LD
CD19	NHL	111	CR (14 months) 40% OS (12 months) 49%	JULIET [74]	LD

## Abbreviations

ALL	acute lymphoblastic leukemia
AML	acute myeloid leukemia
APC	antigen-presenting cell
BCMA	B-cell maturation antigen
CAF	cancer-associated fibroblasts
CAR	chimeric antigen receptor
CI	checkpoint inhibitor
CLL	chronic lymphocytic leukemia
CNS	central nervous system
CR	complete remission
DC	dendritic cell
DLBCL	diffuse large B-cell lymphoma
ECM	extracellular matrix
FDA	Food and Drug Administration
GD2	disialoganglioside
GvHD	graft-versus-host disease
HER2	human epidermal growth factor receptor 2
HL	Hodgkin’s lymphoma
HLA	human leukocyte antigen
IDH	isocitrate dehydrogenase
IDO	indoleamine-2,3-dioxygenase
IDOi	indoleamine 2,3-dioxygenase inhibitor
IFNγ	interferon-gamma
IL	interleukin
ITAM	immunoreceptor tyrosine-based activation motif
JAK1	Janus kinase 1
LAG3	lymphocyte activation gene 3
LD	lymphodepletion
MART1	melanoma antigen recognized by T-cells 1
MDSC	myeloid derived suppressor cell
MHC	major histocompatibility complex
MM	multiple myeloma
MPM	malignant pleural mesothelioma
NGS	next generation sequencing
NHL	В-cell non-Hodgkin lymphoma
NK	natural killer
NKT	natural killer T
NKR	natural killer receptor
NSCLC	non-small-cell lung carcinoma
OR	objective response
OS	overall survival
PBMC	peripheral blood mononuclear cells
PD	progressive disease
PD-1	programmed cell death protein 1
PD-L1	programmed death-ligand 1
PFS	progression-free survival
PI3K	phosphoinositide 3-kinase
PR	partial remission
PSCA	prostate stem cell antigen
PSMA	prostate-specific membrane antigen
PTEN	phosphatase and tensin homolog
R-CHOP	Rituximab, Cyclophosphamide, Hydroxydaunorubicin, Oncovin, Prednisone or Prednisolone
RF	reading frame
R/R	relapsed and refractory
scFv	single-chain variable fragment
SD	stable disease
SNP	single nucleotide polymorphism
synNotch	synthetic Notch
TAM	tumor-associated macrophages
TAP1 –	transporter associated with antigen processing 1
TCM	central memory T-cell
TCR	T-cell receptor
TGFβ	transforming growth factor beta
TGFβRII	TGFβ receptor II
TIL	tumor infiltrating lymphocyte
TMB	tumor mutational burden
TME	tumor microenvironment
TNF	tumor necrosis factor
Treg	regulatory CD4+ T-cells
TRUCK	T-cells redirected for universal cytokine killing
TSCM	stem cell memory T-cell
VEGF	vascular endothelial growth factor
UCAR-T	universal chimeric antigen receptor T-cell
WT1	Wilms tumor protein
